# *Apocynum venetum* Leaf Extract Exerts Antidepressant-Like Effects and Inhibits Hippocampal and Cortical Apoptosis of Rats Exposed to Chronic Unpredictable Mild Stress

**DOI:** 10.1155/2018/5916451

**Published:** 2018-01-16

**Authors:** Ting Wu, Xiangting Li, Tingting Li, Min Cai, Zhonghai Yu, Jingsi Zhang, Zhennian Zhang, Wen Zhang, Jun Xiang, Dingfang Cai

**Affiliations:** ^1^Department of Integrative Medicine, Zhongshan Hospital, Fudan University, Shanghai 200032, China; ^2^Shanghai University of Traditional Chinese Medicine, Shanghai 201203, China

## Abstract

We investigated the effects of* Apocynum venetum* leaf extract (AVLE) on depressive behaviors and neuronal apoptosis in a chronic unpredictable mild stress (CUMS) rat model of depression. Rats were randomly divided into six groups: control, chronic unpredictable mild stress, fluoxetine, AVLE30, AVLE60, and AVLE120. Except for the control group, all rats were submitted to chronic unpredictable mild stress paradigms for four weeks to induce depressive behavior. Neuronal apoptosis was assessed by the terminal deoxynucleotidyl transferase- (TDT-) mediated dUTP-biotin nick end-labeling (TUNEL) method. The expression levels of apoptosis-related proteins, such as B-cell lymphoma 2 (Bcl-2), Bcl-2 Associated X Protein (Bax), cysteine-aspartic acid protease-3 and protease-9 (caspase-3 and caspase-9), cytochrome c (cyt-C), brain-derived neurotrophic factor (BDNF), and cAMP-response element binding (CREB) protein, were evaluated by western blot. Treatment with AVLE (60 or 120 mg/kg/day) significantly improved depressive behavior. Increased apoptosis of hippocampus and cortical neurons were observed in CUMS rats, while 120 mg/kg/day of AVLE significantly reversed these changes and achieved the best antidepressant-like effects among the doses tested. Moreover, AVLE (120 mg/kg) significantly increased Bcl-2, BDNF, and CREB protein expression and decreased Bax, cyt-C, and caspase family protein expression. Our results indicate that AVLE has potent antidepressant activity, likely due to its ability to suppress neuronal apoptosis.

## 1. Introduction

Depression is a type of mood disorder which has a significant influence on social harmony. Clinical manifestations include low mood, anhedonia, sleep disorders and cognitive disorders, and suicidal tendencies. Most antidepressants are effective in only 50% of patients [1], which implies that the monoamine neurotransmitter hypothesis is only part of the pathogenesis of depression, and the mechanisms of antidepressant efficacy remain unknown. Recent studies indicate that neuronal apoptosis may be involved in the pathogenesis and treatment of depression. Apoptosis plays a key role in tissue homeostasis, and considerable evidence suggests that apoptosis and the molecular mechanisms underlying cell death and survival are highly relevant to depression. Depression is associated with impairments in structural plasticity and cellular atrophy [2], and volume decreases have been observed in limbic, hippocampal, and prefrontal cortical brain regions [3]. Postmortem analyses indicate a reduction in neuronal cell body size and increased neural apoptosis in patients with depression [4, 5]. In rodent stress paradigms, stress-induced neuroinflammation, apoptosis, and reduced neurogenesis upregulate apoptosis in the cortex and hippocampus [6]. Antidepressant treatment is proposed to reverse some of these changes [7].

The apoptotic process is programmed and controlled by the balance between pro- (Bax) and anti- (Bcl-2) apoptotic proteins within the cell [8]. In response to stress, these proteins regulate the release of cyt-C, which causes activation of the caspase family of cysteine proteases, widely known as the principal mediators of apoptosis. Among the suspected susceptibility genes involved in the etiology of depression, evidence from clinical and basic research suggests a dysregulation of the pleiotropic transcription factor cAMP response element binding protein (CREB), and one of its target genes, brain-derived neurotrophic factor (BDNF) [9, 10]. CREB is involved in a wide range of neuroplasticity processes, including neuronal apoptosis, which is mediated partly by the expression of BDNF. Chronic antidepressant treatments have been shown to upregulate CREB activity and the expression of BDNF. Interestingly, BDNF also contributes to reverse stress-induced depressive behaviors in female mice subjected to chronic unpredictable stress [11].

The clinical application of* Apocynum venetum* leaf extract (AVLE) in the treatment of various disorders has a long history in Asian countries. AVLE contains many active ingredients, such as rutin, hyperoside, isoquercitrin, astragaloside, and quercetin [12], and its extensive pharmacological effects have been confirmed.* In vitro*, AVLE was reported to exert antihypertensive properties [13] and protect endothelial cells against H_2_O_2_-induced apoptosis [14, 15].* In vivo*, AVLE had a protective effect against ischemic reperfusion injury in rat models [16, 17] and was shown to exhibit hepatoprotective effects against acetaminophen-induced liver injury in mice [18, 19]. Moreover, recent findings indicate that AVLE exhibits antianxiety [20, 21] and antidepressant activity [22–25]. Studies have also shown that AVLE improved depression behaviors in mice, regulated levels of monoamine and dopamine, and increased expression of BDNF [24, 25].* In vitro*, AV extract (25, 50, and 100 *μ*g/mL) suppressed the apoptosis of corticosterone-treated PC12 cells and upregulated BDNF expression [26]. The current research on the antidepressant effects of AVLE has focused on the observation of depressive behavior, and the antidepressant mechanisms have not been thoroughly explored. In our previous study, we demonstrated that AVLE decreased neuronal apoptosis in an* in vitro* model of ischemia-reperfusion [27]. Therefore, in the current study we hypothesized that AVLE may exert antidepressant effects via the inhibition of neuronal apoptosis using the chronic unpredictable mild stress rat model of depression.

## 2. Materials and Methods

### 2.1. Materials

*Apocynum venetum* leaf extract was obtained from dried leaves as previously described [27]. Briefly,* Apocynum venetum* leaves (100 g) were refluxed for 1 h in aqueous ethanol (70%, v/v, 60 mL) twice, and the combined alcoholic extract evaporated to dryness (28 g). The extract (13.5 g) was dissolved in hot water (200 mL) and the pH adjusted to 3.0 with sulfuric acid and then filtered. The filtrate underwent chromatography on a Diaion HP-20 (3.6 cm i.d. × 18 cm; Sigma-Aldrich, St. Louis, MO, USA) column and eluted with water (200 mL) followed by aqueous ethanol (70%, v/v, 200 mL). The aqueous ethanol fraction was collected and evaporated to dryness to obtain AVLE (4.2 g). Fluoxetine hydrochloride (FLX) was obtained from Sigma (St. Louis, MO, USA). Sucrose was purchased from Sigma-Aldrich (St. Louis, MO, USA).

### 2.2. Animals

60 male Wistar rats, 6–8 months old, were purchased from Shanghai University of Traditional Chinese Medicine. Rats were acclimated to their environment for 1 wk prior to the experimental period. Food and water were available ad libitum. After one wk, rats were randomly divided into two groups: control (*n* = 10) and the chronic unpredictable mild depression group (*n* = 50). The control group was housed in groups of 4-5 per cage with a constant 12-h light/dark cycle (lights on/off at 07:00/19:00). The depression model group was housed individually and treated with unpredictable chronic mild stress. All experimental procedures were approved by the Animal Care and Use Committee of the Shanghai University of Traditional Chinese Medicine (Animal Experiment License: SZY201612004).

### 2.3. Chronic Unpredictable Mild Stress Protocol

Model group rats were submitted to 4 wk of CUMS. Modifications to the traditional CUMS protocol were made based on the Willner review [28]. The following stresses were induced randomly: food deprivation, water deprivation, black and white reversal, strobe illumination, white noise, overcrowding, restraint, wet bedding, and cold water swimming. Animals were exposed to two stressors each day in random order, for the duration of the experiment. The effectiveness of the CUMS paradigm was confirmed using the sucrose preference test, open field test, and forced swimming test.

### 2.4. Experimental Procedure

**Figure 1:**
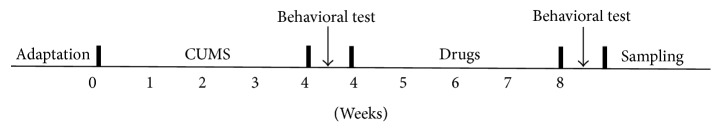
Experimental timeline.

Based on the sucrose preference test results and body weight, rats were divided into six groups after four wk CUMS: control group (distilled water, i.g.), CUMS-vehicle group (distilled water, i.g.), CUMS-fluoxetine group (10 mg/kg, i.g.), CUMS-AVLE30 group (30 mg/kg, i.g.), CUMS-AVLE60 group (60 mg/kg, i.g.), and CUMS-AVLE120 group (120 mg/kg, i.g.). Fluoxetine, which is presently recognized as a classic antidepressant, was used as a positive drug control. Doses were chosen based on previous reports [22]. All agents were administered in a volume of 0.1–0.2 mL once daily between the hours of 10:00 AM and 12:00 AM for 4 wk. The rats were euthanized at the end of the 4 wk treatment under isoflurane anesthesia (2.5–3%). Before and after stress and treatment, rats underwent a battery of behavioral tests aimed at assessing the level of anhedonia (sucrose preference test (SPT)), the emotional response to a novel environment (open field test (OFT)), and depressive-like behaviors (forced swimming test (FST)). A detailed experimental procedure is shown in Figure 1.

### 2.5. Behavioral Testing

Except for SPT, behavior was tracked, recorded, and analyzed using the Etho-Vision system (Noldus Information Technology, Wageningen, The Netherlands). To eliminate interference, OFT and FST were conducted in a dark, soundproof environment. There were 6 rats in each group.

#### 2.5.1. Sucrose Preference Test

The rats were first acclimated by the presence of two bottles of water for 24 h, and then two bottles of 1% sucrose solution for 24 h after the water bottles. Following acclimation, rats were deprived of water for 18 h and then presented with two bottles, one full of water and the other full of 1% sucrose solution, for 1 h. The position of the water and sucrose bottles in the cage was rotated after 30 min to prevent the effect of place preference on drinking behavior. Bottles were weighed prior to and immediately following the test, and the amounts of water and sucrose consumed were measured. The percentage of sucrose preference was calculated based on the following formula: % Sucrose preference = Sucrose consumption/(Sucrose + Water consumption) × 100 [29].

#### 2.5.2. Open Field Test

The OFT was used to evaluate the spontaneous locomotor activity of mice (the total moved distance reflects level of motion; the duration spent in the center area reflects level of anxiety). Rats were placed individually in the middle of an open field apparatus in a plastic box (68 × 68 × 50 cm). Following 1 min of acclimatization to the apparatus, the total distance moved and duration in the center area were recorded over a 5-minute period. After each trial, the open field apparatus was thoroughly cleaned [30].

#### 2.5.3. Forced Swim Test

Two swimming sessions were conducted: a 15 min pretest on the first day followed by a 6 min test the next day. Rats were forced to swim in a plastic cylinder (45 cm high, 20 cm in diameter) filled with fresh water (25 ± 1°C) at a height of 30 cm. The total duration of immobility (s) was measured during the last 4 min of a single 6 min test session. Rats were considered immobile when they made no attempts to escape and engaged only in the movements necessary to keep the head above water. The data were analyzed by two observers blind to the experimental conditions. After the procedure, the animals were dried briefly and returned to their housing cages. The procedure did not cause any loss of life or changes in body weight compared with control rats.

### 2.6. TUNEL Assay

TUNEL was performed per manufacturer protocol (In Situ Cell Death Detection Kit, POD, Roche, Penzberg, Germany), with subtle modifications. Briefly, fixed brain slices were sectioned at 8 *μ*m. Cryosectioned brain slices were rewarmed at room temperature (RT) for 30 min. Brain slices were then washed three times with PBS for 5 min and incubated for 30 min at 37°C with 10% normal goat serum containing 0.3% Triton X-100. Then slices were rinsed three times with PBS for 5 min and incubated in the dark for 2 h in a humidified container at RT with a TUNEL reaction mixture containing the following: 5 *μ*L enzyme solution (terminal deoxynucleotidyl transferase in storage buffer); 50 *μ*L label solution (fluorescein-nucleotide mixture in reaction buffer). Slices were then washed three times in PBS, stained with 4′,6-diamidino-2-phenylindole (DAPI) for 15 min at RT and then washed three times in PBS. Slices were then air-dried and directly observed under a fluorescence microscope (DP71, Olympus). At least 5 randomly selected microscopic fields were used for counting the TUNEL-positive cells (200x magnification). Cell counting was conducted by two investigators blinded to group conditions. There were 6 rats in each group.

### 2.7. Western Blotting

Protein extracts were isolated from hippocampal tissue using lysis buffer [50 mmol/L Tris HCl (pH 7.2) containing 1% sodium deoxycholate, 1% NP-40, 0.15 mmol/L NaCl, and 0.1% sodium dodecyl sulphate (SDS)] (Roche Applied Science, Mannheim, Germany). Bicinchoninic acid assays were performed to measure protein concentrations (Beyotime, Haimen, Jiangsu, China). Equal amounts of protein were loaded to the gel. All samples were separated using sodium dodecyl sulfate polyacrylamide gel electrophoresis (SDS-PAGE) gels and transferred to polyvinylidene difluoride (PVDF) membranes (0.22 *μ*m, Millipore, Billerica, MA, USA) following electrophoresis. The membranes were blocked with 5% non-fat milk at RT for 1 h and incubated at 4°C overnight with the following antibodies: rabbit polyclonal anti-Bcl-2 (1 : 1000, Abcam), rabbit monoclonal anti-Bax (1 : 1000, CST), rabbit monoclonal anti-BDNF (1 : 5000, Abcam), rabbit monoclonal anti-CREB (1 : 1000, CST), rabbit polyclonal anti-caspase-3 (1 : 1000, CST), rabbit monoclonal anti-caspase-9 (1 : 1000, CST), rabbit monoclonal anticytochrome c (1 : 1000, CST), and rat monoclonal anti-glyceraldehyde 3-phosphate dehydrogenase (GAPDH; 1 : 1000, CST). After being incubated with HRP-conjugated antibodies, the bands were visualized using enhanced chemiluminescence (ECL) kits (Millipore, Billerica, MA, USA). Mouse monoclonal anti-GAPDH antibody was used as a loading control. Quantitative analysis of the results was carried out by densitometry scanning of the films, and the data were analyzed by ImageJ. Significant differences between means were statistically assessed by ANOVA. Significant differences between animals were assessed by Dunnett's test. There were 6 rats in each group.

### 2.8. Statistical Analysis

Statistical analysis was performed using GraphPad Prism version 5.0 (GraphPad Prism 5.0, GraphPad Software Inc., San Diego, CA). Western blot data were analyzed by one-way analysis of variance with Dunnett's test. The TUNEL-positive cell count was analyzed using the rank-sum test. All other data were analyzed by one-way ANOVA or Student's *t*-test. A value of *P* < 0.05 was considered significant.

## 3. Results

### 3.1. Effect of AVLE on Depressive Behavior in Rats

**Figure 2:**
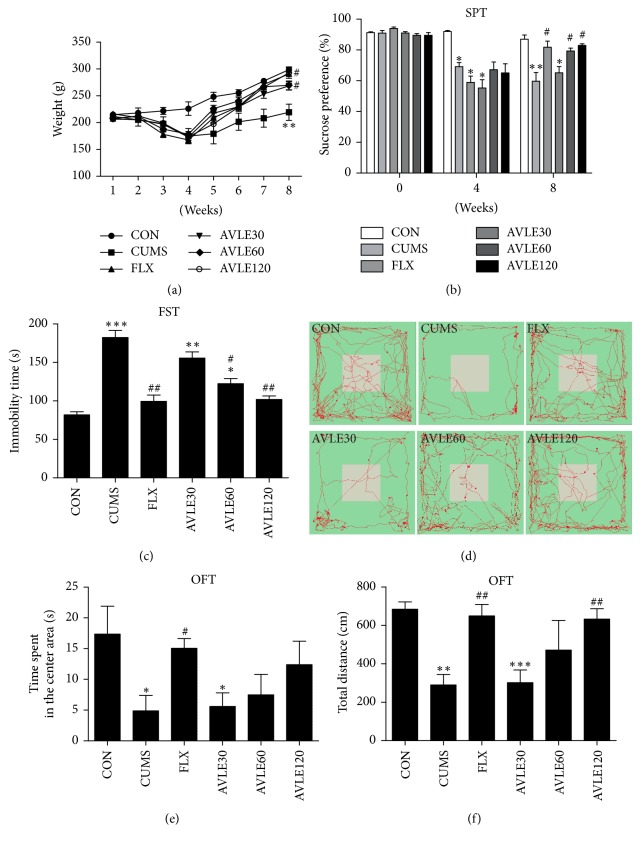
Effect of AVLE on depressive behavior in CUMS rats. (a) Effect of AVLE administration on body weight of CUMS rats. (b) Effect of AVLE administration on sucrose preference in CUMS rats. (c) Effect of AVLE administration on immobility time during forced swimming test in CUMS rats. (d) Trace plot of open field test. (e) Effect of AVLE administration on time spent in the center area during open field test in CUMS rats. (f) Effect of AVLE administration on total distance moved during open field test in CUMS rats. Data are expressed as mean ± SEM (*n* = 6); ^*∗*^*P* < 0.05, ^*∗∗*^*P* < 0.01, and ^*∗∗∗*^*P* < 0.001 compared with the control group; ^#^*P* < 0.05 and ^##^*P* < 0.01 compared with the CUMS group. CON: control; CUMS: chronic unpredictable mild stress; FLX: fluoxetine; AVLE30:* Apocynum venetum* leaf extract (30 mg/kg); AVLE60:* Apocynum venetum* leaf extract (60 mg/kg); AVLE120*: Apocynum venetum* leaf extract (120 mg/kg).

A decrease (22.6%) in body weight was observed in rats after 4 wk of unpredictable mild stress (*P* < 0.01, SEM = 14.036860, *n* = 6). However, a 4 wk treatment of FLX (10 mg/kg/d) and AVLE (120 mg/kg/d) reversed this change (*P* < 0.5) (Figure 2(a)). In the sucrose preference test (Figure 2(b)), we observed that CUMS decreased the consumption of sucrose in rats 23–37%, where FLX (10 mg/kg/d) and AVLE (60, 120 mg/kg/d) significantly increased sucrose preference in CUMS rats (*P* < 0.5). CUMS significantly prolonged the immobility time (122.8%) of rats in FST *P* < 0.001 (SEM = 9.165434, *n* = 6). A significant decrease in the immobility time was elicited by the administration of FLX (10 mg/kg/d) and AVLE (60, 120 mg/kg/d) after 4 wk of treatment in CUMS rats (*P* < 0.01, *P* < 0.05, and *P* < 0.01, resp.) (Figure 2(c)). In the OFT, CUMS induced a marked decrease in the time spent in the center area (71.9%, *P* < 0.05, SEM = 10.76000451, *n* = 6) and total distance moved (57.5%, *P* < 0.01, SEM = 133.9486498, *n* = 6) compared to the control group (Figures 2(d), 2(e), and 2(f)). The total distance moved and the time spent in the center area were increased by simultaneous treatment with FLX (*P* < 0.01, *P* < 0.05) and AVLE120 (*P* < 0.01). These results indicated that the optimum dose of AVLE was 120 mg/kg/d, so the effects of this dose were further analyzed.

### 3.2. Effect of AVLE on Hippocampal and Cortical Neuronal Apoptosis

**Figure 3:**
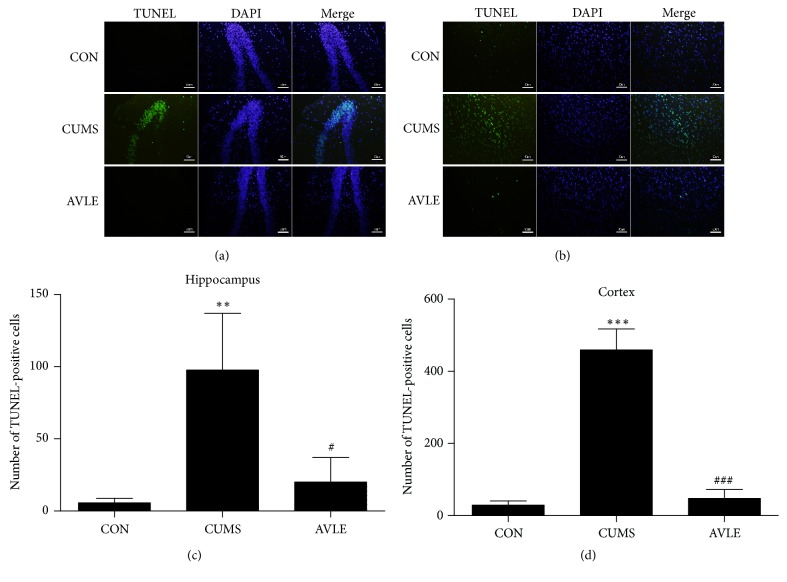
Effect of AVLE on neuronal apoptosis of hippocampus and cortex of CUMS rats. (a, b) TUNEL staining in the hippocampus DG region and cortex of CUMS rats. (c, d) Quantification of TUNEL-positive cells in the hippocampus and cortex. Scale bars: 50 *μ*m. Data are presented as the mean ± SEM (*n* = 6); ^*∗∗*^*P* < 0.01 and ^*∗∗∗*^*P* < 0.001 compared with the control group; ^#^*P* < 0.05 and ^###^*P* < 0.001 compared with the CUMS group. CON: control; CUMS: chronic unpredictable mild stress; AVLE:* Apocynum venetum* leaf extract (120 mg/kg); TUNEL: terminal deoxyribonucleotidyl transferase- (TDT-) mediated dUTP-digoxigenin nick end labeling.

CUMS significantly induced neuronal apoptosis in the hippocampus (the number of increased TUNEL-positive cells = 92, *P* < 0.01, SEM = 27.75588026, *n* = 6) and cortex (the number of increased TUNEL-positive cells = 430, *P* < 0.001, SEM = 41.02912245, *n* = 6) compared with the control group (Figure 3), while AVLE (120 mg/kg/d) attenuated neuronal apoptosis in both the hippocampus and cortex (*P* < 0.05 and *P* < 0.001, resp.).

### 3.3. Effect of AVLE on Apoptosis-Related Proteins, BNDF, and CREB Expression in the Hippocampus

**Figure 4:**
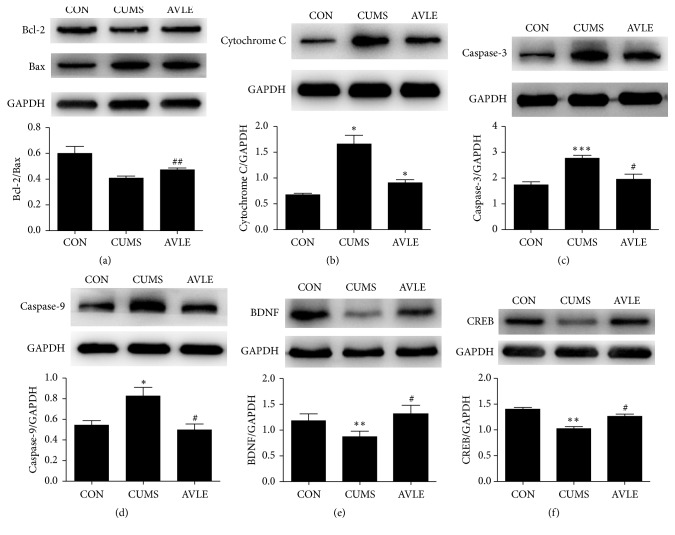
Effect of AVLE on Bcl-2, Bax, cytochrome c, caspase-3, caspase-9, BDNF, and CREB protein expression in the hippocampus of CUMS rats. Representative images of immunoblots are shown in the upper panels. Values in bar graphs are expressed as mean ± SEM (*n* = 6); ^*∗*^*P* < 0.05, ^*∗∗*^*P* < 0.01, and ^*∗∗∗*^*P* < 0.001 compared with the control group; ^#^*P* < 0.05 and ^##^*P* < 0.01 compared with the CUMS group. CON: control; CUMS: chronic unpredictable mild stress; AVLE:* Apocynum venetum* leaf extract (120 mg/kg).

CUMS resulted in a decreased ratio of Bcl-2/Bax expression in the hippocampus. This decrease was reversed by AVLE treatment (120 mg/kg/d) (*P* < 0.01). The expression of apoptosis-related proteins was significantly increased in the CUMS rats versus the control group: cytochrome c expression increased 147.4% (*P* < 0.05), caspase-3 expression increased 59.2% (*P* < 0.001), and caspase-9 expression increased 51.3% (*P* < 0.05). These increases were significantly inhibited by AVLE treatment (120 mg/kg/d) (Figures 4(b), 4(c), and 4(d)). BDNF and CREB expression were also decreased by CUMS compared with the control group: BDNF expression decreased by 26.7% (*P* < 0.01) and CREB expression decreased by 26.9% (*P* < 0.01) but was inhibited by AVLE at 120 mg/kg (both *P* < 0.05) (Figures 4(e) and 4(f)).

## 4. Discussion

*Apocynum venetum* L. (“Luobuma” in Chinese) has long been used as a health tea of the “hypotensor type” in China, Japan, and other Asian countries. However, recent findings indicate that the* Apocynum venetum* leaf also possesses antidepressant activities. For example, an extract of* Apocynum venetum* leaf (30–125 mg/kg) markedly shortened the immobility time of male rats in a forced swimming test (FST). This effect was comparable to that of the tricyclic antidepressant imipramine (20 mg/kg) [22]. In addition, intragastric administration of a flavonoid extract of* Apocynum venetum* leaf (50 and 100 mg/kg) significantly reduced the immobility time of mice in both the forced swimming test and tail suspension test. AVLE has also been shown to increase concentrations of the main neurotransmitters norepinephrine, dopamine, dihydroxyphenyl acetic acid, and homovanillic acid in the hippocampus [25].

The chronic unpredictable mild stress model was developed in the late 1980s by Willner and colleagues and is one of the most extensively utilized animal models of depression. In a typical CUMS scheme, rodents (rats or mice) are subjected to a variety of unpredictable, mild stressors over a sustained period (usually 3-4 wk). The advantage of the CUMS scheme over other depression models is due to the simulation of cardinal symptoms of major depression (face validity), realistic use of inducing conditions (construct validity), and appropriate responsiveness to antidepressant drugs (predictive validity) [28]. Animals will develop a wide spectrum of behavioral, neurobiological, and physiological alterations after stress, which can be effectively reversed by chronic antidepressant treatment [28]. Exposure to a period of stress ultimately leads to the induction of anhedonia (i.e., loss of interest in normally rewarding stimuli), which manifests as a decrease in consumption of sucrose solution.

In the current study, four wk of exposure to CUMS resulted in decreased food intake and weight loss compared to control rats. In addition, SPT, FST, and OFT scores also decreased, indicating the establishment of the model was successful. A range of symptoms, such as loss of appetite, anhedonia, novelty suppression, anxiety, and depression, was observed in the CUMS rats. However, these changes were significantly attenuated following 4 wk of AVLE administration. Treatment with AVLE induced weight gain in the CUMS rats, increased consumption of sucrose solution, time spent in center area and distance moved in the OFT, and decreased immobility time in the FST. Taken together, these results suggest that AVLE has potential antidepressant effects, consistent with previous studies [22, 25].

Structural impairments that regulate mood and cognition, such as decreased size of brain regions (i.e., cortex and hippocampus) and decreased neuronal synapses in these areas, are implicated in depression [2]. Volume decreases have been observed in limbic, hippocampal, and prefrontal cortical brain regions [3]. Postmortem analyses indicate a reduction in neuronal cell body size and increased neural apoptosis in patients with depression [4, 5]. In animal stress models, stress was shown to induce apoptosis in the hippocampus and reduced neurogenesis [31]. These studies suggest that neuronal apoptosis may be involved in the pathogenesis and treatment of depression. The current study demonstrated that neuronal apoptosis was upregulated by CUMS in both the hippocampus and cortical areas in CUMS rats, as shown by TUNEL assay. The expression of proapoptotic proteins (Bax, cytochrome c, caspase-3, and caspase-9) increased, and antiapoptosis protein Bcl-2 expression decreased in response to CUMS. Moreover, a reduction in the expression of BDNF and CREB was observed. After four wk of administration of AVLE (120 mg/kg/d), the observed increase in neuronal apoptosis was significantly reversed, the expressions of cytochrome c, caspase-3, and caspase-9 proteins were inhibited, and the ratio of Bax/Bcl-2 decreased after treatment with AVLE. AVLE also upregulated the expression of BDNF and CREB. Therefore, we conclude that AVLE had an antidepressant effect in rats exposed to CUMS via neuroprotective mechanisms reversal of CUMS-induced neuronal apoptosis in the hippocampus and cortex.

What is the main antidepressant ingredients of* Apocynum venetum* extract? What kinds of ingredients can pass through the blood brain barrier? These may be the future research direction.
